# Effect of Small Molecule on *ex vivo* Expansion of Cord Blood Hematopoietic Stem Cells: A Concise Review

**DOI:** 10.3389/fcell.2021.649115

**Published:** 2021-04-09

**Authors:** Soudeh Ghafouri-Fard, Vahid Niazi, Mohammad Taheri, Abbas Basiri

**Affiliations:** ^1^Department of Medical Genetics, Shahid Beheshti University of Medical Sciences, Tehran, Iran; ^2^Department of Tissue Engineering and Applied Cell Sciences, School of Advanced Technologies in Medicine, Shahid Beheshti University of Medical Sciences, Tehran, Iran; ^3^Urology and Nephrology Research Center, Shahid Beheshti University of Medical Sciences, Tehran, Iran

**Keywords:** small molecules, *ex vivo* expansion, hematopoietic stem cell, expression, mesenchymal stromal cells

## Abstract

Hematopoietic stem cells (HSCs) are a group of cells being produced during embryogenesis to preserve the blood system. They might also be differentiated to non-hematopoietic cells, including neural, cardiac and myogenic cells. Therefore, they have vast applications in the treatment of human disorders. Considering the restricted quantities of HSCs in the umbilical cord blood, inadequate mobilization of bone marrow stem cells, and absence of ethnic dissimilarity, *ex vivo* expansion of these HSCs is an applicable method for obtaining adequate amounts of HSCs. Several molecules such as NR-101, zVADfmk, zLLYfmk, Nicotinamide, Resveratrol, the Copper chelator TEPA, dmPGE2, Garcinol, and serotonin have been used in combination of cytokines to expand HSCs *ex vivo*. The most promising results have been obtained from cocktails that influence multipotency and self-renewal features from different pathways. In the current manuscript, we provide a concise summary of the effects of diverse small molecules on expansion of cord blood HSCs.

## Introduction

Hematopoietic stem cells (HSCs) are a group of cells being produced during embryogenesis to preserve the blood system. Unlike to progenitor cells, these cells have both self-renewal and multipotency. Therefore, HSCs have important applications in the hematopoietic stem cell transplantations (HSCT) and regenerative medicine (Tajer et al., 2019). However, HSCs constitute a minor population of bone marrow cells even less than 0.01% of these cells (Walasek et al., 2012). The fast accessibility and lower need for immune-matching have potentiated the umbilical cord blood as an important source of HSCs for transplantation (Chou et al., 2010). Considering the restricted quantities of HSCs in the umbilical cord blood and inadequate mobilization of bone marrow stem cells (Daniel et al., 2016), *ex vivo* expansion of these HSCs represents an applicable method for obtaining substantial quantities of HSCs. Several strategies have been used to expand these cells among them is addition of several cytokines to the culture media. Yet, this method did not lead to adequate and long lasting expansions (Zhang and Lodish, 2008). Lack of sustained self-renewal and induction of differentiation in HSCs obtained from these protocols (Seita and Weissman, 2010) have restricted the application of these methods. However, more recent studies have attained promising results using hematopoietic expansion medium, comprising cytokines and nutritional complements (Zhang et al., 2019). Other modalities for *ex vivo* expansion of HSCs include co-culture with stromal cells (McNiece et al., 2004), forced over-expression of specific genes (Walasek et al., 2012), and using recombinant proteins for modulation of developmental pathways (Krosl et al., 2003). Besides, lentivirus vectors have been used to deliver a number of genes to enhance engraftment of short term repopulating HSCs (Abraham et al., 2016). Numerous small-sized chemical agents have also been used for such purpose (De Lima et al., 2008; Nishino et al., 2009; Peled et al., 2012). In the current manuscript, we provide a concise summary of the effects of diverse small molecules on expansion of cord blood HSCs.

## StemRegenin-1 (SR-1)

StemRegenin–1 has been shown to enhance expansion of CD34+ hematopoietic progenitors through antagonizing aryl hydrocarbon receptor (Boitano et al., 2010). Co-culture of HSCs with SR-1 and several other factors such as stem cell factor (SCF), FLT-3L, TPO, and IL-6 has resulted to expansion of larger quantities of CD34+ cells (Wagner et al., 2016). In a clinical trial conducted by Wagner et al. (2016) SR–1 has resulted in a 330-fold expansion of CD34+ cells resulting in fast engraftment of neutrophils and platelets in all of assessed patients. According to remarkable effect of this substance on HSCs expansion, non-existence of graft failure and high hematopoietic recovery, SR-1 has been suggested as a solitary agent for HSCT for defeating the major problem of umbilical cord blood transplantation (Wagner et al., 2016).

## Epigenetic Modifiers

Mahmud et al. (2014) have assessed expansion of HSCs when exposed to histone deacetylase (HDAC) inhibitors valproic acid (VPA) and trichostatin A (TSA). These cells were exposed to these agents alone or along with 5-aza-2′-deoxycytidine (5azaD). Their experiment showed the superior effects of VPA on expansion of CD34+CD90+ cells and progenitor cells. *In vivo* studies verified the impacts of VPA on prevention of HSC defects. Besides, combination of 5azaD and TSA resulted in expansion of HSCs that preserve their features through serial transplantation. Expression analysis revealed differential expression of genes participating in the expansion and maintenance of HSCs in 5azaD/TSA- and VPA-treated cells, respectively. Overexpression of quiescence genes by histone acetylation has been suggested as the underlying mechanism of these observations (Mahmud et al., 2014). Saraf et al. (2015) have assess the effects of sequential treatment of CD34+ mobilized human peripheral blood (MPB) with 5azaD and TSA in accompany with cytokines. They observed significant expansion of CD34+CD90+ cells in 5azaD/TSA-treated cells. They also detected over-expression of genes participating in self-renewal in these cells. Such over-expression was accompanied by global hypomethylation (Saraf et al., 2015). Milhem et al. (2004) have treated human bone marrow CD34+ cells with a cytokine cocktail, 5azaD, and TSA. They observed remarkable expansion of a group of these cells. Notably, 5azaD- and TSA-pre-exposed cells but not those treated with cytokines alone preserved the capacity to repopulate NOD mice (Milhem et al., 2004). After a period of cytokine priming, VPA-exposed CD34+ cells have produced CD34+CD90+ multipotent cells. Co-culture of CD34+ cells with combination of cytokines and VPA has resulted in a more remarkable expansion of these cells. Such exposure has led to enhancement of aldehyde dehydrogenase activity, and over-expression of a number of cell surface proteins namely CD90, CD117, CD49f, and CD184. Treatment of CD34+ cells with VPA has led to production of higher quantities of repopulating cells in the immune deficient mice (Chaurasia et al., 2014).

## Nicotinamide

As a suppressor of SIRT1, Nicotinamide has been shown to preclude differentiation and enhance expansion of hematopoietic stem and progenitor cells (HSPCs) (Peled et al., 2012). Horwitz et al. (2014) have used an *ex vivo*–expanded cell product that contains nicotinamide (NiCord) in a phase I clinical trial. They reported total or fractional neutrophil and T cell engraftment in most of patients. Moreover, they reported stability of NiCord engraftment in all individual during the follow-up period. Median neutrophil recovery was faster in individuals transplanted with NiCord (Horwitz et al., 2014).

## Serotonin

The neurotransmitter serotonin has various extraneuronal roles. This substance has been shown to induce megakaryocytopoiesis through 5−HT2 receptors (Yang et al., 1996). In addition, serotonin promotes expansion of CD34+ cells to early stem/progenitors and multilineage committed progenitors. The effects of this agents on repopulating cells in the expansion culture has also been verified in SCID mice suggesting the impact of serotonin in the development of HSCs and modulation of bone marrow niche (Yang et al., 2007).

Table 1 summarizes the results of studies which assessed the effects of small molecules on *ex vivo* expansion of cord blood hematopoietic stem cells.

**TABLE 1:** Effects of small molecules on *ex vivo* expansion of cord blood hematopoietic stem cells.

Molecule	Input cells	Media type and cytokines	Expansion period	Result	Possible mechanism	Reference
SR1	HSC CD34+	–	15 days	330-fold increase in HSC CD34+	Inhibition of AhR and differentiation of HSC	Wagner et al., 2016
5azaD/TSA	HSC CD34+	Serum-containing media, SCF, Flt3L, IL-3, TPO	9 days	10.7-fold increase in HSC CD34+	Hypomethylation of self-renewal genes in HSC and activation of them	Mahmud et al., 2014
DEAB	HSC CD34+	IMDM, 10% FBS, SCF, TPO, Flt3L	7 days	–	Inhibition of aldehyde dehydrogenase (ALDH) and downregulation of retinoic acid signaling pathway and overexpression of cEBP	Chute et al., 2006
P18IN003 and P18IN011	HSC CD34+	IMDM/10% FBS, SCF, TPO, Flt3L	3 days	–	Inhibition of sp18^INK4C^ and increase in self-renewal of HSC	Srikanth et al., 2015
TEPA	HSC CD34+	α-MEM, 10% FCS, SCF, TPO, Flt3L, IL3, IL6	21 days	–	Increase in the activity of cytochrome c oxidase	Prus and Fibach, 2007
Nicotinamide	HSC CD34+	–	–	104-fold increase in HSC CD34+	Inhibition of SIRT1 deacetylase and differentiation of HSC	Horwitz et al., 2014
Notch ligand	HSC CD34+	–	16 days	164-fold increase in HSC CD34+	Induction of self-renewal by activation of Notch receptor	Dahlberg et al., 2011
MSC co-culture	Unselected	–	11 days	30.1-fold increase in HSC CD34+	Increase in SDF-1 which diminishes differentiation of HSC	Robinson et al., 2006
Nicotinamide	HSC CD34+	–	21 days	72-fold increase in HSC CD34+	Inhibition of SIRT1 deacetylase and differentiation of HSC	Horwitz et al., 2014
Fucosylation	Unselected	–	30 min	NA	Increase in the homing ability of HSC	Popat et al., 2015
5azaD/TSA	HSC CD34+	Serum-containing media, SCF, Flt3L, IL-3, TPO	9 days	3.6-fold increase in HSC CD34+	Hypomethylation of self-renewal genes in HSC and activation of them	Saraf et al., 2015
5azaD/TSA	HSC CD34+	Serum-containing media, SCF, Flt3L, IL-3, TPO	9 days	2.5-fold increase in HSC CD34+	Hypomethylation of self-renewal genes in HSC and activation of them	Milhem et al., 2004
VPA	HSC CD34+	Serum-containing media, SCF, Flt3L, IL-3, TPO	9 days	64.6-fold increase in HSC CD34+	Overexpression of quiescence genes by histone acetylation	Mahmud et al., 2014
SB203580	HSC CD133+	SCF, TPO, Flt3L	7 days	–	Decline in senescence of HSC, overexpression of CXCR4	Zou et al., 2012
OAC1	HSC CD34+	RPMI, 10% FBS, SCF, TPO, Flt3L	4 days	–	Overexpression of HOXB4 gene, Induction of OCT4 expression	Huang et al., 2016
dm-PGE2	HSC CD34+ and CD133+	IMDM/2% FCS	3, 6, or 9 h	–	Increase in homing, survival, and proliferation of HSCs	Simsek et al., 2010
VPA	HSC CD34+	Serum-free media, 16 h priming with SCF, Flt3L, IL-3, TPO	7 days	20-fold increase in HSC CD34+	Overexpression of pluripotency genes	Chaurasia et al., 2014
VPA	HSC CD34+	Serum-containing media, SCF, Flt3L, IL-3, TPO	7 days	89-fold increase in HSC CD34+	Overexpression of pluripotency genes	Chaurasia et al., 2014
UM171	HSC CD34+	Fed-batch culture system	7 days	60–75-fold increase in HSC CD34+	Suppression of erythroid and megakaryocytic differentiation	Fares et al., 2014
Notch ligand	HSC CD34+	Serum-free media, SCF, Flt3L, IL-3, IL-6, TPO	17–21 days	222-fold increase in HSC CD34+	Stimulation of Notch signaling and induction of self-renewal	Delaney et al., 2010
SR1	HSC CD34+	Serum-free media, SCF, TPO, IL-6, Flt3L	21 days	270-fold increase in HSC CD34+	Inhibition of AhR and differentiation of HSC	Boitano et al., 2010
5azaD, TSA	HSC CD34+	Serum-containing Media, SCF, TPO, FL, IL-3	9 days	5-fold increase in HSC CD34+	Unclear	Araki et al., 2006
UNC0638	HSC CD34+	Serum-free media, SCF, TPO, Flt3L, IL-6	14 days	60-fold increase in HSC CD34+	Unclear	Chen et al., 2012
NR-101	HSC CD34+	StemSpan/SCF, TPO, Flt3L	7 days	–	Activation of TPO receptor (c-MPL), activation of c-MPL, STAT5 Pathways, overexpression of VEGF	Nishino et al., 2009
zVADfmk	HSC CD34+	StemPro/SCF, TPO, Flt3L, IL6	10 days	–	Pan caspase inhibitor, overexpression of Bcl-2 and downregulation of Caspase-3, Activation of notch1	Kale and Limaye, 2010
Nicotinamide	HSC CD34+	α-MEM/10% FBS, SCF, TPO, Flt3L, IL6	21 days	–	Downregulation of p53 gene	Peled et al., 2012
Resveratrol	HSC CD34+	StemSpan/SCF, TPO, Flt3L, IL6	9 days	–	Overexpansion of CD34+/CD133+ and cell cycle associated genes, downregulation of cyclooxygenase 1 (COX-1)	Heinz et al., 2015
Copper chelator (TEPA)	HSC CD133+	–	21 days	6-fold increase in HSC CD133+	Unclear	De Lima et al., 2008
dmPGE2	Unselected	–	2 h	NA	Increasing Wnt signaling	Hagedorn et al., 2014
Garcinol	HSC CD34+	StemSpan/SCF, TPO, Flt3L	7 days	–	Inhibition of HATs	Nishino et al., 2011
Serotonin (5-HT)	HSC CD34+	QBSF-60/SCF, TPO, Flt3L, IL6	8 days	–	Activation of 5-HT receptor, Down-regulation of Caspase-3	Yang et al., 2007

*TEPA, Tetraethylenepentamine; 5azaD/TSA, 5aza-2′- Deoxycytidine/Trichostatin A; MSC, Mesenchymal Stromal Cells; VPA, Valproic Acid; FCS, fetal calf serum; dmPGE2, Dimethyl-Prostaglandin E2; Notch, Notch ligand; HSC, Hematopoietic Stem Cell; SR1, StemRegenin 1; TSA, trichostatin A; AhR, aryl hydrocarbon receptor; SCF, stem cell factor; SIRT1, Silent Mating Type Information Regulation 2 Homolog 1; IL-6, interleukin 6; TPO, thrombopoietin; SDF1, Stromal Derived Factor-1; DEAB, diethylaminobenzaldehyde; IMDM, Iscove’s Modified Dulbecco’s Medium; FBS, fetal bovine serum; RPMI, Roswell Park Memorial Institute; VEGF, Vascular endothelial growth factor; α-MEM, Minimum Essential Medium Eagle – alpha modification; HAT, histone acetyltransferases.*

## Discussion

*Ex vivo* expansion of HSCs obtained from umbilical cord blood has become a major research field because of its application in the treatment of several hematological disorders (Flores-Guzmán et al., 2013). This process is expected to mimic the normal bone marrow niche which is consisted of stromal cells and their produced materials. Thus, nearly all expansion protocols contain early- and late-acting cytokines to enhance growth of hematopoietic cells. Moreover, various kinds of stromal cells such as primary MSCs can improve the efficiency of expansion. Several promising results have been obtained from these experiments leading to design of nearly optimal protocols. Different combinations of cytokines can promote commitment of hematopoietic progenitor cells (HPCs) from certain lineages, i.e., erythroid or myeloid ones. Thus, selection of appropriate cytokines is important in reaching the optimal yield. The possibility of differentiation of HSCs to non-hematopoietic cells, including neural, cardiac and myogenic cells have expanded their applications in the treatment of human disorders (Flores-Guzmán et al., 2013). Moreover, expanded HSC have been used in clinical trials to inhibit Graft-versus-host disease (GVHD) following HSCT. Examples of these clinical trials are those conducted by Nohla Therapeutics^1^ and exciting trials by ExCellThera^2^. The latter has received Food and Drug Administration orphan drug designation for ECT-001. ECT-001 comprises a small molecule, UM171, and an adjusted culture method for expansion of HSC. The primary results of this trial have been promising in reduction of risk of GVHD^3^.

Small molecules represent novel modalities for *ex vivo* expansion of cord blood HSCs. These molecules have also been used for the purpose of expansion of HSPCs with primitive phenotype. Screening of a patented library containing tens of small molecules has shown the necessity of including SCF, thrombopoietin and Fms-related tyrosine kinase 3 ligand in the cocktail for enhancing expansion of these cells in addition to C7. Moreover, adding insulin like growth factor binding protein 2 to the cocktail slightly increased expansion (Bari et al., 2016).

The efficacy of these molecules is different in various aspects of HSCT. For instance, SR-1, Notch-ligand and nicotinamide-based methods has been associated with fast neutrophil recovery (Delaney et al., 2010; Horwitz et al., 2014; Wagner et al., 2016). Epigenetic modifiers such as VPA are among the mostly assessed agents in *ex vivo* expansion studies. Such agents increase expression of multipotency and self-renewal genes mainly through global modifications in epigenetic marks.

Small molecules exert their effects through different routes including induction of expression of pluripotency genes, modulation of methylation pattern of self-renewal genes and regulation of retinoic acid, Wnt, and Notch signaling pathways. Inhibition of apoptosis is another putative mechanism of participation of small molecules in *ex vivo* expansion of HSCs, as the caspase and calpain inhibitors have been proved to be effective in this regard (Kale and Limaye, 2010). Such strategy has been proved to be effective in enhancement of efficacy of transplantation in animal models, since temporary up-regulation of prosurvival BCL-XL as considerably promoted survival and engraftment of HSCs and progenitor cells (Kollek et al., 2017). In some cases, the exact molecular mechanism of their impacts on HSCs is not clear. Identification of such mechanisms would facilitate design of optimal protocols and avoidance of possible side-effects of associated therapies.

As a rule, strong expansion of HSPCs with sustaining engraftment requires the synergistic functions of several enhancers of HSC expansion (Pineault and Abu-Khader, 2015). This fact has been reflected in better therapeutic results and enhanced engraftment in clinical trials that used the synergistic effects of cytokines and HSPC expansion enhancers (Pineault and Abu-Khader, 2015). Therefore, application of combination of small molecules and cytokines is a suggested strategy for expansion of HSCs and inhibition of their differentiation (Wang et al., 2017). The underlying mechanism of such observations might be the effect of small molecules in modulation of regulatory roles of cytokines on signaling pathways. The results of *in vitro* studies have been verified in a number of experiments using irradiated NOD/SCID mice (Eldjerou et al., 2010; Budak-Alpdogan et al., 2012). These types of studies would help in the evaluation of cell viability and engraftment capacity *in vivo*. Functional consequences of HSC expansion strategies in the clinical settings have been assessed through different methods. The time to neutrophil or platelet engraftments, enhancement of patients’ survival, occurrence of GVHD, infectious conditions, or relapse rates are among parameters which have been recognized as clinically relevant parameters in this regard (Kiernan et al., 2017). The most important parameter seems to be patients’ survival. It is worth mentioning that in spite of important developments in *ex vivo* expansion of HSCs, the capability to follow HSC fate decisions within the bone marrow microenvironment is inadequate (Papa et al., 2020).

Another application of small-molecules-induced expansion of HSCs is that transcriptome analysis of these cells can facilitate identification of the crucial pathways and molecules of HSC self-renewal, thus providing novel target molecules for *in vitro* expansion of these cells (Zhang et al., 2020).

Taken together, several molecules such as SR-1, NR-101, the caspases inhibitor zVADfmk, the calpain inhibitor zLLYfmk, Nicotinamide, Resveratrol, the Copper chelator TEPA, dmPGE2, Garcinol, and serotonin have been used in combination of cytokines to expand HSCs *ex vivo*. The most promising results have been obtained from cocktails that influence multipotency and self-renewal features from different pathways. These cocktails include several growth factors, molecules that regulate signaling pathways and vectors that manipulate expression of genes. Putative target genes in this regard are HOX genes and cell cycle associated genes as well as those participating in Wnt, Notch, and Shh pathways. Each constituent of these cocktails might affect some aspects of multipotency and self-renewal, constructing a synergic system to yield the best results. These elements should enhance self-renewal, suspend differentiation, promote homing, and suppress apoptosis of HSCs (Zhang and Gao, 2016). Precise assessment of the expansion aptitude of cell populations through quantification of the generated cells using immunophenotypic methods or functional assessments is an important requirement of comparison studies for identification of best *ex vivo* expansion methods. Aptitude of expansion and preservation of multipotent HPCs can be precisely assessed by the numbers of lineage specific colony-forming units and colony-forming unit mix and Human Long-Term Culture-Initiating Cell (LTC-IC) activity (Ali et al., 2014). Concomitant conduction of these methods is crucial for identification the expanded lineage in each experimental condition.

As different methods have been used for such quantifications, it is not easy to precisely compare the results of different studies. Most mentioned studies has used CD34+ HSCs as input cell population. Yet, a number of studies lave used unselected cell populations which complicate the comparison studies due to different extents of enrichment for primitive cells.

Considering the complexity of natural HSC milieu and existence of a wide range of cells and their produced molecules in addition to secreted cytokines, application of multifaceted strategies seems to yield more promising results. Examples of such strategies are co-culture of stromal cells with other niche components including extracellular matrix proteins (Deutsch et al., 2010; Celebi et al., 2011) or Sonic hedgehog (Bhardwaj et al., 2001) and induction of expression of genes which promote self-renewal (Watts et al., 2010; Aguila et al., 2011).

Finally, documentation of expansion of actual HSCs rather than HPCs is an important prerequisite of clinical applications although both cell population have their own clinical uses. Such important step has been missed in some early studies in this field.

## Author Contributions

MT and SG-F wrote the draft of the manuscript and revised it. AB and VN designed the tables and collected the data. All authors approved the submitted version.

## Conflict of Interest

The authors declare that the research was conducted in the absence of any commercial or financial relationships that could be construed as a potential conflict of interest.

## Footnotes

1 https://clinicaltrials.gov/ct2/show/NCT01690520

2 https://www.clinicaltrials.gov/ct2/show/NCT04103879

3 https://www.insidertracking.com/excellthera-receives-fda-orphan-drug-designation-for-ect-001-for-the-prevention-of-graft-versus-host-disease
